# Genome-wide identification and expression analysis of the *AT-hook Motif Nuclear Localized* gene family in soybean

**DOI:** 10.1186/s12864-021-07687-y

**Published:** 2021-05-18

**Authors:** Min Wang, Bowei Chen, Wei Zhou, Linan Xie, Lishan Wang, Yonglan Zhang, Qingzhu Zhang

**Affiliations:** 1 College of Life Sciences, Northeast Forestry University, Harbin, 150040 People’s Republic of China; 2 Key Laboratory of Saline-Alkali Vegetative Ecology Restoration, Ministry of Education, College of Life Science, Northeast Forestry University, Harbin, 150040 China; 3 State Key Laboratory of Tree Genetics and Breeding, Northeast Forestry University, Harbin, 150040 People’s Republic of China

**Keywords:** AT-hook motif, PPC domain, AHL, Gene family, Soybean

## Abstract

**Background:**

Soybean is an important legume crop and has significant agricultural and economic value. Previous research has shown that the *AT-Hook Motif Nuclear Localized* (*AHL*) gene family is highly conserved in land plants, playing crucial roles in plant growth and development. To date, however, the *AHL* gene family has not been studied in soybean.

**Results:**

To investigate the roles played by the *AHL* gene family in soybean, genome-wide identification, expression patterns and gene structures were performed to analyze. We identified a total of 63 *AT-hook motif* genes, which were characterized by the presence of the AT-hook motif and PPC domain in soybean. The *AT-hook motif* genes were distributed on 18 chromosomes and formed two distinct clades (A and B), as shown by phylogenetic analysis. All the AHL proteins were further classified into three types (I, II and III) based on the AT-hook motif. Type-I was belonged to Clade-A, while Type-II and Type-III were belonged to Clade-B. Our results also showed that the main type of duplication in the soybean *AHL* gene family was segmented duplication event.

To discern whether the *AHL* gene family was involved in stress response in soybean, we performed cis-acting elements analysis and found that *AHL* genes were associated with light responsiveness, anaerobic induction, MYB and gibberellin-responsiveness elements. This suggest that *AHL* genes may participate in plant development and mediate stress response. Moreover, a co-expression network analysis showed that the *AHL* genes were also involved in energy transduction, and the associated with the gibberellin pathway and nuclear entry signal pathways in soybean. Transcription analysis revealed that *AHL* genes in Jack and Williams82 have a common expression pattern and are mostly expressed in roots, showing greater sensitivity under drought and submergence stress. Hence, the *AHL* gene family mainly reacts on mediating stress responses in the roots and provide comprehensive information for further understanding of the *AT-hook motif* gene family-mediated stress response in soybean.

**Conclusion:**

Sixty-three *AT-hook motif* genes were identified in the soybean genome. These genes formed into two distinct phylogenetic clades and belonged to three different types. Cis-acting elements and co-expression network analyses suggested that *AHL* genes participated in significant biological processes. This work provides important theoretical basis for the understanding of *AHLs* biological functions in soybean.

## Background

The *AT-Hook Motif Nuclear Localized (AHL)* gene family is highly conserved across all land plants, and the AHL transcription factors were previously described in mosses and flowering plants [1]. It has been previously demonstrated that some conserved transcription factor families were essential to plant growth and stress tolerance during plant evolution, including the *bHLH* and *NAC* gene families [2–7]. However, some of the transcription factor families that have played important roles in plants evolution remain understudied. The *AT-hook motif* gene family is highly conserved across plant species and plays relevant roles during plant development.

The *AT-hook motif* gene family is involved in in very important biological processes in plants. For example, *AHL* genes are associated with the regulation of plant reproductive development and the formation of ears in maize [8]. In rice, the *DP1* gene, encoding for an AT-hook DNA binding protein, plays an important role in flower development [9]. Moreover, the *AT-hook motif* gene family is also able to regulates the expression of cell-specific genes. The overexpression of the *GIANT KILLER(GIK)* gene, which encodes an AHL protein, leads to serious defects in the reproductive organs and the reduction of expression levels in associated genes [10]. In *Arabidopsis*, the *AHL* gene *BoMF2* is preferentially expressed in the stamens and its overexpression results in a significantly shorter siliques and a decrease in pollen vigor relative to the wild type [11]. Importantly, the *AHL* gene family also has been identified to regulate hormone balance in plants, especially gibberellin [12], jasmonic acid and auxin-related genes [13–15]. This is also illustrated by previous transcriptomic analysis showing that *AtAHL13* is a key factor regulating jasmonic acid biosynthesis signal transduction and pathogen immunity [16]. Importantly, AHL proteins also can regulate the chromatin state. The AT-hook motif protein AHL22 regulates flowering time by interacting with the deacetylase at the FLOWERING LOCUS site. The overexpression of *AHL22* in Arabidopsis mutant exhibits delayed flowering, significantly decreased transcription activity and acetylation of histone H3 at the FLOWERING LOCUS, and to an increased demethylation rate of H3 Lysine 9 [17]. It has also been previously reported that the protein TEK (TRANSPOSABLE ELEMENT SILENCING VIA AT-HOOK) protein, which is encoded by an *AHL* gene, is involved in the regulation of silent TEs. Specifically, knocking down of TEK leads to increased histone acetylation and decreased H3K9me2 and DNA methylation levels in the target loci [18]. Recently, a total of 37 *AHL* genes have been identified in maize. The transcription levels in different tissues suggest that AHL proteins are involved in maize pollen development, drought response and senescence [19]. A high number of 48, 51, 99 *AHL* genes also be found in different three cotton genomes, and gene expression analysis indicated that the majority of *AHL* genes in Clade-B were expressed in the stem whereas the Clade-A genes were expressed in the ovules [20]. Furthermore, the 20 *AHL* genes uncovered in rice exhibited three expression patterns, all *OsAHL* genes may be functional genes with 3 different expression patterns [21]. The overexpression *OsAHL1* improved rice response to multiple stress tolerances, especially drought resistance [22].

These studies suggest that the *AT-hook motif* gene family not only plays important roles in plant growth and development of plants, but also affects plant response to stress and hormonal stimulus. These studies still lack a systematic investigation on how the *AT-hook motif* gene family regulates plant stress. Hence, this study evaluated plant response to drought and submergence stress mediated by *AHL* genes.

AHL proteins contain two conserved domains, the AT-hook motif and the plant and Prokaryote Conserved (PPC) domain, also known as the Domain of Unknown Function#296 (DUF296) [23]. The PPC domain contains 120 amino acids, and has the same secondary or tertiary structure from prokaryotes to higher plants [23]. The hydrophobic region at the C-terminus of the PPC domain plays an important role in nuclear location and protein interaction [1, 24], indicating that AHLs may have a role in regulating plant transcriptional activity [25]. The AT-hook motif contains one or two conserved Arg-Gly-Arg motifs that are used to bind the AT-rich DNA regions. This result has been confirmed in both prokaryotes and eukaryotes organisms, including the High Mobility Group A (HMGA) proteins in mammals [24]. The binding of the AT-hook motif to the AT-rich DNA forms a concave structure and results in insertion of two arginines [26]. So the *AT-hook motif* gene family regulates plant growth and development through DNA-protein interoperability and the formation of protein-homo/hetero-trimeric complex [25, 26].

Phylogenetic analysis of land plants showed that the AHL proteins can be divided into two categories based on differences in the PPC domain, Clade A and Clade B [1]. The conserved amino acid sequence of Clade A is Leu-Arg-Ser-His, whereas the equivalent in Clade B is Phe-Thr-Pro-His [1]. Nonetheless, the amino acid sequence Gly-Arg-Phe-Glu-Ile-Leu is sometimes part of the PPC domain and is essential for the function of some AHL proteins [25]. The differences of AT-hook motif make it possible to classify AHL proteins into three different types (I, II, and III). Type-I belongs to Clade-A, Type-II and Type-III belong to Clade-B. The AT-hook motif of Type-I has a Gly-Ser-Lys-Asn-Lys conserved sequence at the C-terminal of the Arg-Gly-Arg center, while Types II and III instead contain Arg-Lys-Tyr. In angiosperms, phylogenetic analysis allowed to divide Clades A and B into five and four subfamilies, respectively [1]. The observed similar expression patterns in each clade suggest that *AHLs* retained their biological functions in the course of evolution [1].

Soybean (*Glycine max L*. Merr) is the major leguminous species and an important source of protein worldwide, playing a vital role in human survival and development [27]. The function of the proved *AT-hook motif* genes provides the basis for our research and the detailed genome-wide analysis of the *AT-hook motif* gene family in soybean has been not performed. In this study according to the findings of the *AT-hook motif* gene family in maize and cotton, we annotated the *AT-hook motif* gene family in the soybean genome and identified 63 *AHL* genes. We then analyzed function of these genes and respective protein structure features, as well as their chromosome locations, gene duplication events, Gene Ontology annotations, phylogenetic relationships, collinear co-expression network and expression patterns. Our results will foster understanding of the biological functions of the *AHL* family in soybean.

## Results

### Phylogenetic analysis of the *AT-hook motif* gene family in soybean

**Fig. 1:**
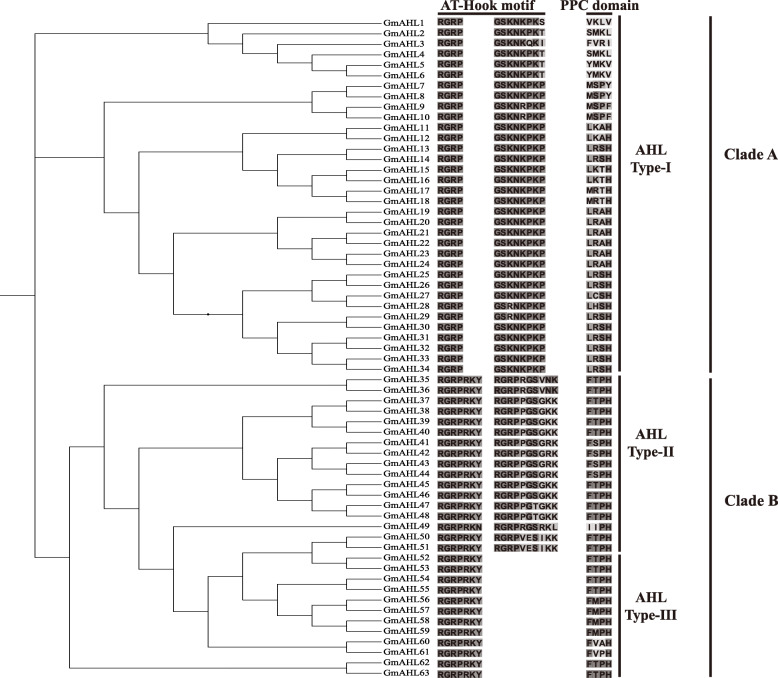
Phylogenetic analysis of the soybean AHL proteins. The obtained phylogenetic tree is shown on the left, with the conserved domain is displayed on the right

We predicted a total of 63 AHL proteins containing the AT-hook motif and PPC domain in soybean, named GmAHL1 ~ GmAHL63 (Fig. 1, Table 1). To infer the evolution relationship among the AHL proteins in soybean, phylogenetic analysis was performed on the full-length AHL protein sequences. Our results showed that AHL proteins in soybean can be divided into two clades, Clade-A (with 34 proteins) and Clade-B (with 29 proteins), as previously described in other land plants [1]. Multiple sequence alignments allowed to further divide, Clade-A and Clade-B into Type-I (54%), Type-II (27%) and Type-III (19%). The higher abundance of Type I in soybean is also consistent with observations in other land plants [1], and shows that AHL proteins are conserved in the course of evolution.

**Table 1:** The length and the position of the *AT-hook motif* gene family of chromosomes

Type	Gene	Gene accession NO.	Gene Location	Gene Length	CDS Length	Protein Length	PI	MW
AHL TypeI	*GmAHL1*	Glyma.20G038600	Chr20:5985361..5985945	585	585	194	8.84	20,706.77
	*GmAHL2*	Glyma.20G039500	Chr20:6424264..6425299	1036	519	172	6.96	18,273.72
	*GmAHL3*	Glyma.20G040100	Chr20:6927297..6928210	914	768	255	7.9	27,471.58
	*GmAHL4*	Glyma.07G230900	Chr07:41176872..41177633	762	762	258	9.42	27,562.06
	*GmAHL5*	Glyma.20G039200	Chr20:6293233..6293943	711	711	236	9.19	25,372.55
	*GmAHL6*	Glyma.20G039300	Chr20:6354437..6355147	711	711	236	8.79	25,263.36
	*GmAHL7*	Glyma.06G093400	Chr06:7353687..7356232	2546	855	284	6.79	29,680.28
	*GmAHL8*	Glyma.04G091600	Chr04:8052537..8054787	2251	843	280	6.59	29,126.71
	*GmAHL9*	Glyma.14G181200	Chr14:44412425..44413662	1238	771	256	8.95	27,181.59
	*GmAHL10*	Glyma.02G213500	Chr02:39966501..39967977	1477	816	271	7.78	28,325.73
	*GmAHL11*	Glyma.14G028600	Chr14:2074152..2074901	750	750	249	9.33	26,365.24
	*GmAHL12*	Glyma.02G285500	Chr02:46650504..46652113	1610	747	248	8.79	26,208.95
	*GmAHL13*	Glyma.03G022700	Chr03:2358393..2360007	1615	933	310	6.59	32,357.99
	*GmAHL14*	Glyma.01G144400	Chr01:47862376..47864806	2431	867	288	7.11	29,581.95
	*GmAHL15*	Glyma.01G213100	Chr01:54443421..54445622	2202	903	300	6.30	30,910.32
	*GmAHL16*	Glyma.11G028800	Chr11:2073771..2076640	2870	897	298	6.34	31,034.59
	*GmAHL17*	Glyma.05G054200	Chr05:4921245..4923175	1931	852	283	6.19	29,746.22
	*GmAHL18*	Glyma.17G136600	Chr17:11034761..11036699	1939	864	287	6.19	30,264.73
	*GmAHL19*	Glyma.18G247200	Chr18:53457034..53458586	1553	807	268	5.66	27,850.01
	*GmAHL20*	Glyma.09G245800	Chr09:46779198..46781547	2350	813	270	5.44	28,184.34
	*GmAHL21*	Glyma.01G198800	Chr01:53270493..53271245	753	753	250	6.1	26,278.35
	*GmAHL22*	Glyma.11G043100	Chr11:3156212..3156964	753	753	250	5.86	26,240.41
	*GmAHL23*	Glyma.17G155400	Chr17:13134432..13135858	1427	756	251	8.54	27,140.41
	*GmAHL24*	Glyma.05G111500	Chr05:29729388..29730984	1597	831	276	6.21	29,364.85
	*GmAHL25*	Glyma.18G036200	Chr18:2830848..2832883	2036	909	302	5.54	32,201.29
	*GmAHL26*	Glyma.11G221200	Chr11:31641566..31645035	3470	870	289	5.7	30,635.88
	*GmAHL27*	Glyma.14G066800	Chr14:5511222..5513114	1893	714	237	4.90	24,853.35
	*GmAHL28*	Glyma.02G249800	Chr02:43733046..43736212	3167	690	229	4.62	23,864.19
	*GmAHL29*	Glyma.10G167100	Chr10:40144743..40146501	1759	843	280	6.13	29,230.44
	*GmAHL30*	Glyma.20G222000	Chr20:45695377..45696210	834	834	277	5.98	28,749.99
	*GmAHL31*	Glyma.10G008400	Chr10:812787..815045	2259	813	270	5.41	27,464.43
	*GmAHL32*	Glyma.20G087200	Chr20:32632218..32634457	2240	807	268	5.49	27,411.30
	*GmAHL33*	Glyma.20G202300	Chr20:43941717..43944283	2567	912	303	8.73	30,926.49
	*GmAHL34*	Glyma.10G188400	Chr10:42143305..42144254	950	873	290	6.06	29,511.80
AHL TypeII	*GmAHL35*	Glyma.06G014600	Chr06:1098115..1101942	3828	1068	355	10.16	36,559.94
	*GmAHL36*	Glyma.04G014600	Chr04:1119416..1123175	3760	1074	357	10.41	36,813.52
	*GmAHL37*	Glyma.05G111800	Chr05:29745228..29750532	5305	1089	362	9.19	36,729.08
	*GmAHL38*	Glyma.17G155200	Chr17:13112585..13118577	5993	1071	356	9.41	36,028.69
	*GmAHL39*	Glyma.11G042900	Chr11:3139534..3143800	4267	1020	253	8.81	26,256.53
	*GmAHL40*	Glyma.01G198900	Chr01:53282978..53287009	4032	1017	338	9.1	35,208.29
	*GmAHL41*	Glyma.01G219600	Chr01:54903061..54907533	4473	1074	357	9.73	36,504.56
	*GmAHL42*	Glyma.11G023900	Chr11:1720878..1725368	4491	1059	352	9.89	35,948.07
	*GmAHL43*	Glyma.05G207300	Chr05:38947662..38951376	3715	1059	352	9.64	36,082.51
	*GmAHL44*	Glyma.08G014000	Chr08:1080565..1085103	4539	1059	352	9.68	36,040.37
	*GmAHL45*	Glyma.03G011200	Chr03:1079855..1087560	7706	1023	340	9.69	34,658.14
	*GmAHL46*	Glyma.07G072300	Chr07:6560938..6567765	6828	1023	340	9.77	34,917.49
	*GmAHL47*	Glyma.09G260600	Chr09:47883584..47890792	7209	1026	341	9.86	35,155.54
	*GmAHL48*	Glyma.18G231300	Chr18:51979095..51987062	7968	1029	342	9.82	35,223.57
	*GmAHL49*	Glyma.11G189800	Chr11:26216330..26220334	4005	1113	370	6.07	38,502.16
	*GmAHL50*	Glyma.10G178000	Chr10:41125424..41132741	7318	993	330	7.73	34,728.24
	*GmAHL51*	Glyma.20G212200	Chr20:44876238..44882406	6169	993	330	6.55	34,643.13
AHL TypeIII	*GmAHL52*	Glyma.09G153600	Chr09:37642252..37648087	5836	1035	344	8.36	35,572.19
	*GmAHL53*	Glyma.16G204400	Chr16:36534047..36539263	5217	1035	344	7.82	35,775.53
	*GmAHL54*	Glyma.05G053800	Chr05:4865327..4870695	5369	984	327	9.04	33,433.79
	*GmAHL55*	Glyma.17G136200	Chr17:10982415..10988350	5936	996	331	9.34	34,087.76
	*GmAHL56*	Glyma.01G143100	Chr01:47640893..47648188	7296	1041	346	9	35,718.2
	*GmAHL57*	Glyma.03G023500	Chr03:2486917..2493916	7000	1041	346	9	35,740.29
	*GmAHL58*	Glyma.09G268900	Chr09:48639768..48644136	4369	1014	337	9.25	34,996.59
	*GmAHL59*	Glyma.18G220900	Chr18:50788395..50793712	5318	1017	284	9.55	29,606.77
	*GmAHL60*	Glyma.10G065500	Chr10:6273279..6277937	4659	1191	396	5.82	41,543.51
	*GmAHL61*	Glyma.13G150600	Chr13:26410180..26415049	4870	1140	379	6.76	39,672.25
	*GmAHL62*	Glyma.03G251800	Chr03:44744746..44751071	6326	1041	346	9.04	36,513.61
	*GmAHL63*	Glyma.19G249200	Chr19:49523295..49529220	5926	1086	361	9.04	38,142.25

We found that Clade-A, which contained the conserved PPC domain sequences Leu-Arg-Ser-His and Leu-Arg-Ala-His, was more variable than Clade-B, with a PPC domain comprised of Phe-Thr-Pro-His. At the same time, we also observed that the variability of the PPC domain in soybean AHL proteins is higher than that of maize [19]. It is possible that the increase in PPC domain variability may extend the range of biological functions of AHL proteins.

The Type-I AT-hook motif contains four conserved conservative amino acid residues at the N-terminus of Arg-Gly-Arg-Pro, and eight conserved amino acid residues at the C-terminus of Gly-Ser-Lys-Asn-Lys-Pro-Lys-Pro. This contrasts with an observed seven and ten conserved amino acid residues at the N-terminal and C-terminal of Type II, respectively. Comparing the structure of Type-III and Type-II, they have the same PPC domain and the N-terminal of AT-hook motif conservative structure, but the former lack conserved amino acids residues of AT-hook motif at the C-terminal. The observed diversity in the AT-hook motif and PPC domains across soybean AHL proteins are likely to result in diverse biological functions.

### Gene structure and motif prediction analysis in the *AT-hook motif* gene family in soybean

**Fig. 2:**
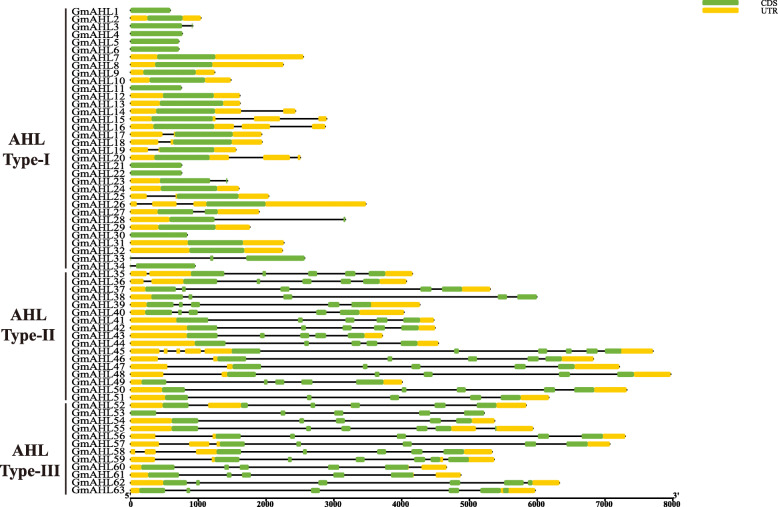
Gene structure analysis of the *AT-hook motif* gene family in soybean. The x-axis shows the inferred length of the different genes (5′ to 3′) and their respective CDS (green) and UTR (yellow)

We implemented a gene structure analysis and estimated the length of AHL genes, and the variability in the number of CDS and UTRs (Fig. 2, Table 1). The length of the *AHL* gene family ranges from 585 bp to 7968 bp, with a total of 12 genes (mostly from Clade A), lacking the UTR, and some showing a variable number of introns and exons (usually Types II and III showed a higher number of introns). Type-I genes were the shortest and contained the lowest number of CDS, which began to increase from Glyma.20G202300. Among them, Type-II and Type-III have two or more introns, which are more obvious than Type-I. Thus, we believe that Type-II and Type-III evolved from Type-I. This result is consistent with the report of maize *AHL* gene family [19]. In eukaryotes, introns and exons alternately form genes. In plants, up to 60% of the genes undergo splicing, most of which occurs in introns [28]. After the introduction of intron-mediated enhancement(IME) into Arabidopsis, mRNA accumulation increased by 24 times and the activity of the reporter enzyme increased by 40 times, indicating that introns have an important influence on the regulation of gene expression in plants [29]. This was also observed in maize, where introns increased the expression level of the genes *Zm00001d018515* and *Zm00001d051861* [19]. The alternative splicing of introns results in a diverse range of encoded proteins and thus to abundant biological functions. So it is possible that the increased number of introns in soybean *AHLs* expand the abundance of AHL proteins. In Type-I of maize, only one gene has UTR, while most genes have UTR in soybean [19], indicating that *AHLs* gene structure of different species is diverse. In summary, we suspect that Type-II and Type-III introns enable plants to acquire more complex and diverse biological functions, and at the same time lay the foundation for the further expansion of intron-carrying *AHLs*.

**Fig. 3:**
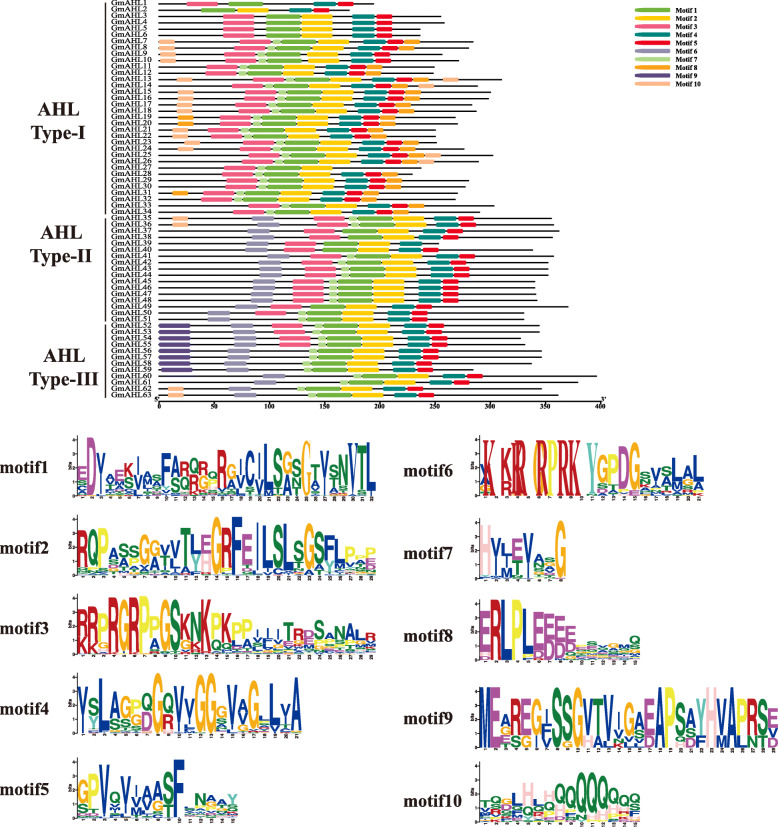
Conservative motif prediction of the *AT-hook motif* gene family. All motifs were identified using the MEME website. A total of ten different motifs are represented by different colors, with the motif sequence shown below. The length of the amino acid was inferred by ruler at bottom. Different colors of letters represent different kinds of amino acids residues, and the size of letters represents the frequency of amino acid occurrence. Most of the genes in the same clade contain the similar motifs

Next, MEME website was used to predict the protein motifs (Fig. 3). We found a total of ten conserved motifs were identified in the AHL proteins (Table 2), which contained of amino acids ranges from 8 to 32 while the sits rang from 8 to 62.

**Table 2:** E-value, Sites Width of *AHLs* conserved motif

	E-value	Sites	Width
motif1	6.0e-1101	62	32
motif2	1.0e-966	62	29
motif3	1.3e-650	50	29
motif4	1.7e-616	62	21
motif5	1.90E-302	61	15
motif6	2.3e-336	29	21
motif7	2.00E-120	52	8
motif8	3.50E-105	25	15
motif9	1.80E-68	8	29
motif10	5.10E-64	20	15

The motifs 3 and 6 had a common conserved Arg-Gly-Arg core, whereby likely belong to the AT-hook motif family. The motif 3 is defined as type I AT-hook motif, and motif 6 is defined as II AT-hook motif. Type-I AHL proteins contains a I AT-hook motif, Type-II contains both I and II AT-hook motifs, and Type-III only has a II AT-hook motif. The sequences downstream of the Arg-Gly-Arg core share common conserved that play an important role in AHL proteins [1]. Interestingly, there is also a conserved sequence Gly-Arg-Phe-Glu-Ile-Leu (motif 2) sequence in the PPC domain. This motif is not only found in soybeans, but also in other land plants, previous study has shown that this motif has an important influence on the PPC domain [1]. It is worth noting that all AHL proteins contain motif 1, motif 4 and motif 5, indicating the consistency of the AHL protein sequences.

In summary, the results of our gene structure and motif prediction analyses indicate that the *AHL* gene family has a consistent and evolutionary diversity in soybean and other land plants [1], including maize [19] and cotton [20].

### Evolution relationship of the *AT-hook motif* gene family in different species

**Fig. 4:**
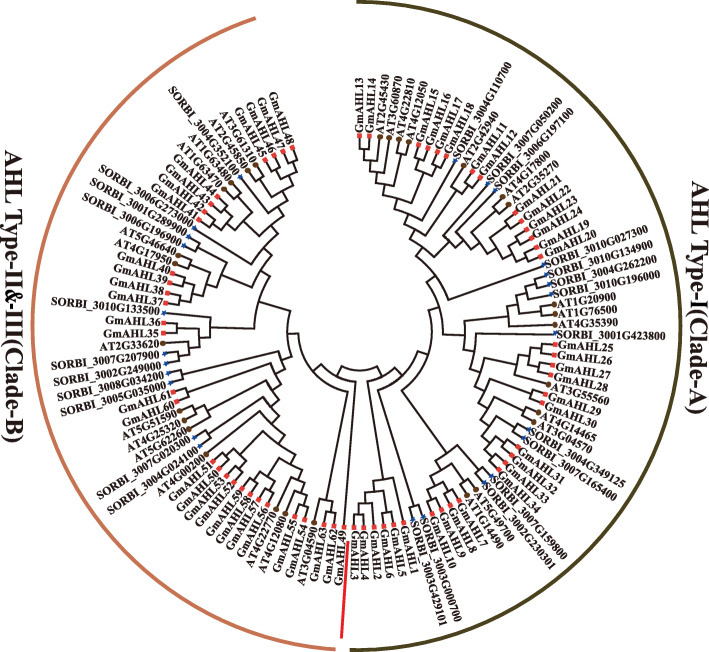
Phylogenetic tree of AHLs in different species (represented by the different colors) using complete protein sequences. We used different colors to represent different species. The red squares represent *Glycine max* L. Merr. The brown circles represent *Arabidopsis thaliana*. The blue stars represent sorghum. Clade-A and clade-B are separated by the red line

In order to further explore the evolutionary relationship between AHLs in different species by selecting *Arabidopsis thaliana*, sorghum (*Sorghum bicolor L*) and soybean as materials and constructing a phylogenetic tree a phylogenetic tree (Fig. 4). Patterns of different colors are used to represent different species. The phylogeny includes 29, 63 and 25 full-length AHL proteins from *Arabidopsis*, soybean and sorghum, respectively. Our analysis showed that the AHL genes of these species can be divided into two distinct clades, A and B. A total of 15 and 14 proteins belonged to Clade-A in *Arabidopsis* and sorghum, respectively, compared to an observed 14 and 11 in Clade-B (Table 3). While Type-I was the more conserved of all types, the lack of a new subgroup between Types II and III in Clade-B indicates the divergence of these proteins occurred relatively late. To sum up, the phylogenetic tree highlights the consistency of the evolution of AHLs among different species, together with the determination of the homology relationships between species provides insights for the future analysis of the biological functions of these proteins.

**Table 3:** The number of *AHLs* in *Arabidopsis*, *Glycine max* and Sorghum

Category	***Arabidopsis***	***Glycine max***	Sorghum
**Clade A**	15	34	14
**Clade B**	14	29	11
**Total number**	29	63	25

### Chromosome location, duplication, GO annotations and collinearity analysis of the *AT-hook motif* gene family in soybean

**Fig. 5:**
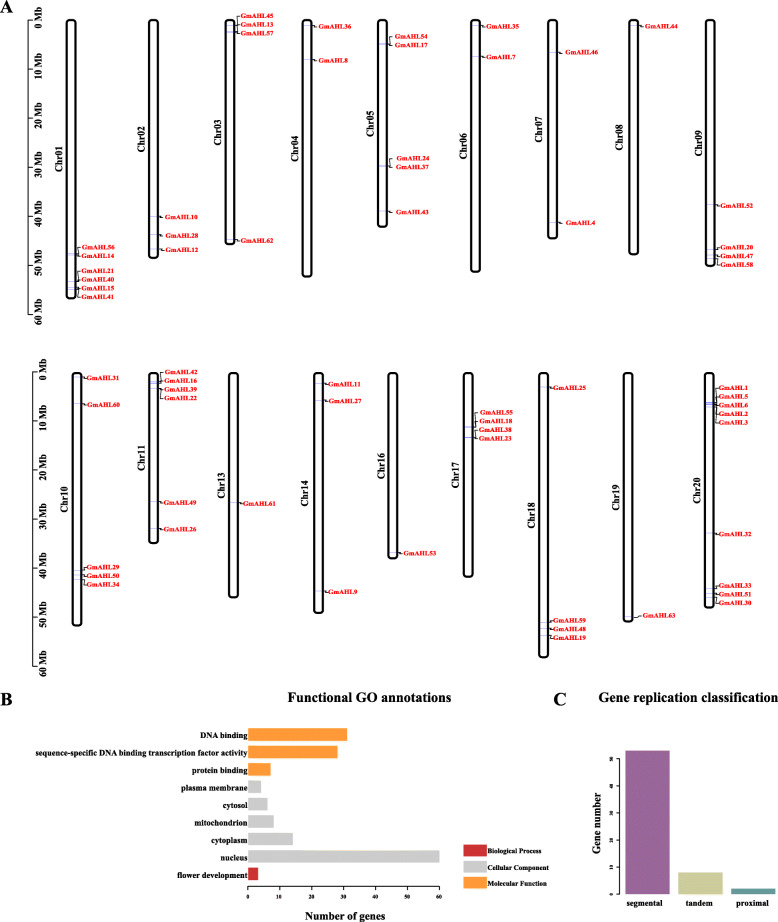
Chromosome location (**a**), functional GO annotations (**b**) and gene replication classification (**c**) of the *AT-hook motif* genes in *Glycine max*. **a** 63 *AT-hook motif* genes were distributed on chromosomes 1–20. The chromosomes number are indicated on the left side of each chromosome representation. The scale of chromosomal length is shown on the left (in Mb). Gene names are indicated by the red letters. **b** Different colors represent different biological processes. **c** Different colors represent different replication types

In order to study the arrangement of 63 *AHL* genes to 20 different chromosomes in the soybean genome (Fig. 5a). The gene location information was in Table 1. Sixty-three *AT-hook motif* genes are distributed on 20 soybean chromosomes. There are 9 *AHLs* on chromosome 20, 1 *AHL* on chromosome 19 and no *AHL* on chromosome 12 and 15. And found that the distribution of these genes on chromosomes was independent of chromosomal length.

In the current study, we then used GO enrichment analysis to predict the potential biological functions of *AHLs*. As shown in Fig. 5b and Table 4, *AHLs* are involved in different biological functions of biological process(BP), molecular functions(MF), and cellular component(CC). Among all the enriched biological functions, we detected an association that the biological process(BP) biological process is related to flowering development, indicating that the *AHL* gene family interfere in the growth and development of floral organs in soybean, which is consistent with the data published in Arabidopsis [17]. As for cellular component is the most abundant, the most of the cell components are located in the nucleus. In terms of the molecular function (MF) category, we identified DNA binding (GO: 0003677), sequence-specific DNA binding transcription factor activity (GO: 0003700) and protein binding (GO: 0005515) are identified. Most AHL proteins evolved to bind DNA and are able to specifically target DNA to perform different biological processes, suggesting AHLs can regulate the expression of other genes.

**Table 4:** The functional annotations of the *AT-hook motif* genes in soybean

Glyma Name	Annotation ID	Description
*GmAHL56*	GO:0005634	nucleus
*GmAHL14*	GO:0005634	nucleus
*GmAHL21*	GO:0005634	nucleus
*GmAHL40*	GO:0005634	nucleus
*GmAHL15*	GO:0005634	nucleus
*GmAHL41*	GO:0005634	nucleus
*GmAHL10*	GO:0005634	nucleus
*GmAHL28*	GO:0005634	nucleus
*GmAHL12*	GO:0005634	nucleus
*GmAHL45*	GO:0005634	nucleus
*GmAHL13*	GO:0005634	nucleus
*GmAHL57*	GO:0005634	nucleus
*GmAHL36*	GO:0005634	nucleus
*GmAHL8*	GO:0005634	nucleus
*GmAHL54*	GO:0005634	nucleus
*GmAHL17*	GO:0005634	nucleus
*GmAHL24*	GO:0005634	nucleus
*GmAHL37*	GO:0005634	nucleus
*GmAHL43*	GO:0005634	nucleus
*GmAHL35*	GO:0005634	nucleus
*GmAHL7*	GO:0005634	nucleus
*GmAHL46*	GO:0005634	nucleus
*GmAHL4*	GO:0005634	nucleus
*GmAHL44*	GO:0005634	nucleus
*GmAHL52*	GO:0005634	nucleus
*GmAHL20*	GO:0005634	nucleus
*GmAHL47*	GO:0005634	nucleus
*GmAHL58*	GO:0005634	nucleus
*GmAHL31*	GO:0005634	nucleus
*GmAHL60*	GO:0005634	nucleus
*GmAHL29*	GO:0005634	nucleus
*GmAHL50*	GO:0005634	nucleus
*GmAHL34*	GO:0005634	nucleus
*GmAHL42*	GO:0005634	nucleus
*GmAHL16*	GO:0005634	nucleus
*GmAHL39*	GO:0005634	nucleus
*GmAHL22*	GO:0005634	nucleus
*GmAHL49*	GO:0005634	nucleus
*GmAHL26*	GO:0005634	nucleus
*GmAHL61*	GO:0005634	nucleus
*GmAHL11*	GO:0005634	nucleus
*GmAHL9*	GO:0005634	nucleus
*GmAHL53*	GO:0005634	nucleus
*GmAHL55*	GO:0005634	nucleus
*GmAHL18*	GO:0005634	nucleus
*GmAHL38*	GO:0005634	nucleus
*GmAHL23*	GO:0005634	nucleus
*GmAHL25*	GO:0005634	nucleus
*GmAHL59*	GO:0005634	nucleus
*GmAHL48*	GO:0005634	nucleus
*GmAHL19*	GO:0005634	nucleus
*GmAHL1*	GO:0005634	nucleus
*GmAHL5*	GO:0005634	nucleus
*GmAHL6*	GO:0005634	nucleus
*GmAHL2*	GO:0005634	nucleus
*GmAHL3*	GO:0005634	nucleus
*GmAHL32*	GO:0005634	nucleus
*GmAHL33*	GO:0005634	nucleus
*GmAHL51*	GO:0005634	nucleus
*GmAHL30*	GO:0005634	nucleus
*GmAHL56*	GO:0005654	nucleoplasm
*GmAHL21*	GO:0005654	nucleoplasm
*GmAHL57*	GO:0005654	nucleoplasm
*GmAHL54*	GO:0005654	nucleoplasm
*GmAHL58*	GO:0005654	nucleoplasm
*GmAHL60*	GO:0005654	nucleoplasm
*GmAHL61*	GO:0005654	nucleoplasm
*GmAHL55*	GO:0005654	nucleoplasm
*GmAHL59*	GO:0005654	nucleoplasm
*GmAHL56*	GO:0005730	nucleolus
*GmAHL57*	GO:0005730	nucleolus
*GmAHL54*	GO:0005730	nucleolus
*GmAHL58*	GO:0005730	nucleolus
*GmAHL60*	GO:0005730	nucleolus
*GmAHL61*	GO:0005730	nucleolus
*GmAHL55*	GO:0005730	nucleolus
*GmAHL59*	GO:0005730	nucleolus
*GmAHL56*	GO:0005737	cytoplasm
*GmAHL21*	GO:0005737	cytoplasm
*GmAHL57*	GO:0005737	cytoplasm
*GmAHL54*	GO:0005737	cytoplasm
*GmAHL24*	GO:0005737	cytoplasm
*GmAHL20*	GO:0005737	cytoplasm
*GmAHL58*	GO:0005737	cytoplasm
*GmAHL60*	GO:0005737	cytoplasm
*GmAHL22*	GO:0005737	cytoplasm
*GmAHL61*	GO:0005737	cytoplasm
*GmAHL55*	GO:0005737	cytoplasm
*GmAHL23*	GO:0005737	cytoplasm
*GmAHL59*	GO:0005737	cytoplasm
*GmAHL19*	GO:0005737	cytoplasm
*GmAHL56*	GO:0005739	mitochondrion
*GmAHL57*	GO:0005739	mitochondrion
*GmAHL54*	GO:0005739	mitochondrion
*GmAHL58*	GO:0005739	mitochondrion
*GmAHL60*	GO:0005739	mitochondrion
*GmAHL61*	GO:0005739	mitochondrion
*GmAHL55*	GO:0005739	mitochondrion
*GmAHL59*	GO:0005739	mitochondrion
*GmAHL40*	GO:0005829	cytosol
*GmAHL36*	GO:0005829	cytosol
*GmAHL37*	GO:0005829	cytosol
*GmAHL35*	GO:0005829	cytosol
*GmAHL39*	GO:0005829	cytosol
*GmAHL38*	GO:0005829	cytosol
*GmAHL45*	GO:0005886	plasma membrane
*GmAHL46*	GO:0005886	plasma membrane
*GmAHL47*	GO:0005886	plasma membrane
*GmAHL48*	GO:0005886	plasma membrane
*GmAHL14*	GO:0009908	flower development
*GmAHL15*	GO:0009908	flower development
*GmAHL13*	GO:0009908	flower development
*GmAHL56*	GO:0003677	DNA binding
*GmAHL21*	GO:0003677	DNA binding
*GmAHL40*	GO:0003677	DNA binding
*GmAHL41*	GO:0003677	DNA binding
*GmAHL12*	GO:0003677	DNA binding
*GmAHL45*	GO:0003677	DNA binding
*GmAHL57*	GO:0003677	DNA binding
*GmAHL62*	GO:0003677	DNA binding
*GmAHL36*	GO:0003677	DNA binding
*GmAHL54*	GO:0003677	DNA binding
*GmAHL37*	GO:0003677	DNA binding
*GmAHL43*	GO:0003677	DNA binding
*GmAHL35*	GO:0003677	DNA binding
*GmAHL46*	GO:0003677	DNA binding
*GmAHL44*	GO:0003677	DNA binding
*GmAHL52*	GO:0003677	DNA binding
*GmAHL47*	GO:0003677	DNA binding
*GmAHL58*	GO:0003677	DNA binding
*GmAHL60*	GO:0003677	DNA binding
*GmAHL42*	GO:0003677	DNA binding
*GmAHL39*	GO:0003677	DNA binding
*GmAHL49*	GO:0003677	DNA binding
*GmAHL61*	GO:0003677	DNA binding
*GmAHL11*	GO:0003677	DNA binding
*GmAHL53*	GO:0003677	DNA binding
*GmAHL55*	GO:0003677	DNA binding
*GmAHL38*	GO:0003677	DNA binding
*GmAHL59*	GO:0003677	DNA binding
*GmAHL48*	GO:0003677	DNA binding
*GmAHL63*	GO:0003677	DNA binding
*GmAHL51*	GO:0003677	DNA binding
*GmAHL14*	GO:0003700	sequence-specific DNA binding transcription factor activity
*GmAHL21*	GO:0003700	sequence-specific DNA binding transcription factor activity
*GmAHL15*	GO:0003700	sequence-specific DNA binding transcription factor activity
*GmAHL10*	GO:0003700	sequence-specific DNA binding transcription factor activity
*GmAHL28*	GO:0003700	sequence-specific DNA binding transcription factor activity
*GmAHL12*	GO:0003700	sequence-specific DNA binding transcription factor activity
*GmAHL13*	GO:0003700	sequence-specific DNA binding transcription factor activity
*GmAHL8*	GO:0003700	sequence-specific DNA binding transcription factor activity
*GmAHL17*	GO:0003700	sequence-specific DNA binding transcription factor activity
*GmAHL24*	GO:0003700	sequence-specific DNA binding transcription factor activity
*GmAHL7*	GO:0003700	sequence-specific DNA binding transcription factor activity
*GmAHL20*	GO:0003700	sequence-specific DNA binding transcription factor activity
*GmAHL31*	GO:0003700	sequence-specific DNA binding transcription factor activity
*GmAHL29*	GO:0003700	sequence-specific DNA binding transcription factor activity
*GmAHL34*	GO:0003700	sequence-specific DNA binding transcription factor activity
*GmAHL16*	GO:0003700	sequence-specific DNA binding transcription factor activity
*GmAHL22*	GO:0003700	sequence-specific DNA binding transcription factor activity
*GmAHL26*	GO:0003700	sequence-specific DNA binding transcription factor activity
*GmAHL11*	GO:0003700	sequence-specific DNA binding transcription factor activity
*GmAHL9*	GO:0003700	sequence-specific DNA binding transcription factor activity
*GmAHL18*	GO:0003700	sequence-specific DNA binding transcription factor activity
*GmAHL23*	GO:0003700	sequence-specific DNA binding transcription factor activity
*GmAHL25*	GO:0003700	sequence-specific DNA binding transcription factor activity
*GmAHL19*	GO:0003700	sequence-specific DNA binding transcription factor activity
*GmAHL2*	GO:0003700	sequence-specific DNA binding transcription factor activity
*GmAHL32*	GO:0003700	sequence-specific DNA binding transcription factor activity
*GmAHL33*	GO:0003700	sequence-specific DNA binding transcription factor activity
*GmAHL30*	GO:0003700	sequence-specific DNA binding transcription factor activity
*GmAHL41*	GO:0005515	protein binding
*GmAHL12*	GO:0005515	protein binding
*GmAHL43*	GO:0005515	protein binding
*GmAHL44*	GO:0005515	protein binding
*GmAHL50*	GO:0005515	protein binding
*GmAHL42*	GO:0005515	protein binding
*GmAHL11*	GO:0005515	protein binding

Gene duplication is a common process in plant evolution that leads to the expansion of gene families, of which tandem and segmental gene duplication events are the most common in angiosperms [30–33]. In order to further examine the evolution of *AHLs* in soybean, we analyzed gene duplication events in the *AT-hook motif* gene family, as shown in Fig. 5c and Table 5. And showed that 84% of *AHL* genes result from segmental duplication events, while 13% represent tandem gene duplication events, and the remaining 3% are proximal. These results suggest that segment duplication events may be the main driver of *AHL* gene family evolution.

**Table 5:** Types of gene replication

Gene Name	Gene Name	Duplication Type
*GmAHL5*	*GmAHL6*	tandem
*GmAHL7*	*GmAHL10*	segmental
*GmAHL8*	*GmAHL7*	segmental
*GmAHL9*	*GmAHL8*	segmental
*GmAHL10*	*GmAHL8*	segmental
*GmAHL11*	*GmAHL12*	segmental
*GmAHL12*	*GmAHL11*	segmental
*GmAHL13*	*GmAHL14*	segmental
*GmAHL15*	*GmAHL17*	segmental
*GmAHL16*	*GmAHL15*	segmental
*GmAHL18*	*GmAHL17*	segmental
*GmAHL21*	*GmAHL24*	segmental
*GmAHL22*	*GmAHL21*	segmental
*GmAHL23*	*GmAHL21*	segmental
*GmAHL24*	*GmAHL22*	segmental
*GmAHL25*	*GmAHL28*	segmental
*GmAHL26*	*GmAHL28*	segmental
*GmAHL27*	*GmAHL28*	segmental
*GmAHL28*	*GmAHL25*	segmental
*GmAHL35*	*GmAHL36*	tandem
*GmAHL37*	*GmAHL39*	segmental
*GmAHL38*	*GmAHL37*	segmental
*GmAHL39*	*GmAHL40*	segmental
*GmAHL40*	*GmAHL38*	segmental
*GmAHL41*	*GmAHL43*	segmental
*GmAHL42*	*GmAHL41*	segmental
*GmAHL43*	*GmAHL44*	segmental
*GmAHL44*	*GmAHL41*	segmental
*GmAHL45*	*GmAHL46*	segmental
*GmAHL54*	*GmAHL55*	segmental
*GmAHL62*	*GmAHL63*	segmental
*GmAHL63*	*GmAHL62*	segmental

**Fig. 6:**
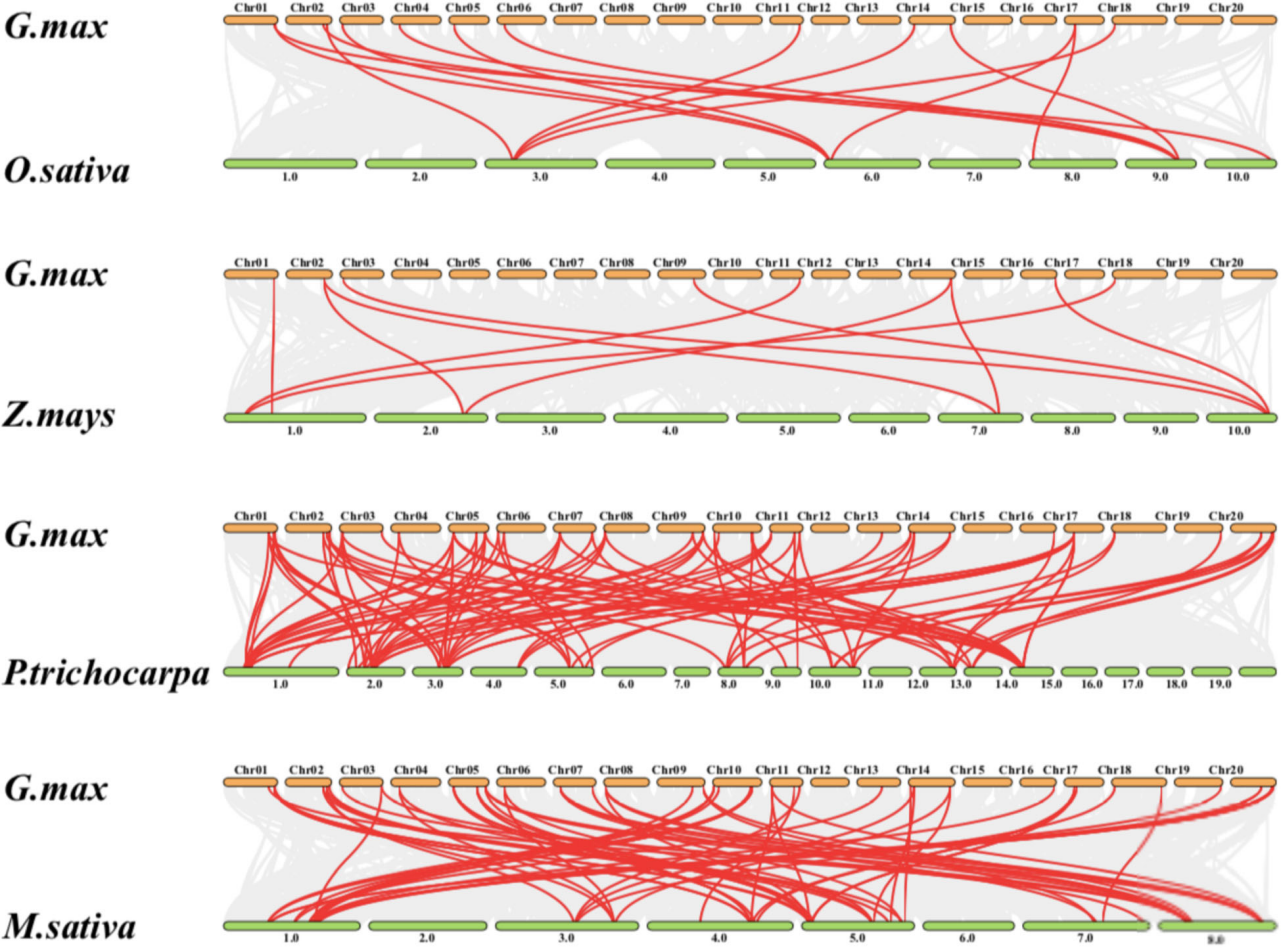
Collinearity analysis of the *AT-hook motif* gene family between *Oryza sativa*, *Populus trichocarpa*, *Medicago sativa*, *Zea mays* and *Glycine max*. The grey lines are indicative to the collinear block within *Glycine max*. The red lines are indicative to the syntenic *AT-hook motif* genes pairs

The collinearity relationship of *AHLs* of two dicotyledonous plants (Poplar and *Medicago*) and two monocots plants (rice and maize) plants were investigated in order to explore the potential evolutionary relationships (Fig. 6). The results revealed a higher homology between soybean, *Medicago* and *Populus* than that between rice and maize. Compared with monocots, more *AHL* homologous genes are found in dicots. Some soybean *AHL* genes are collinear with *AHL* genes in other plants, particularly in *Populus* and *Medicago*, which suggests that these genes may play important roles in plant evolution. These results can be useful for subsequent comparative studies of *AHL* genes with known functions.

### Promoter sequence analysis of the *AT-hook motif* gene family in soybean

**Fig. 7:**
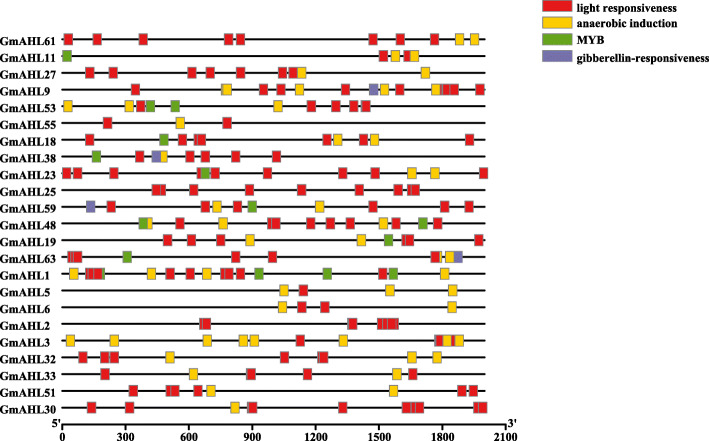
The cis-acting elements of the promoter sub-region. The four elements contained in the *AT-hook motif* gene family include light responsiveness, anaerobic induction, MYB and gibberellin-responsiveness elements. Different colors represent different elements

In organisms, the gene promoter region is located upstream of genes, binds to transcription factors is called the cis-regulatory element, which plays an important role in the biological regulation of gene expression under stress [34]. We identified cis-regulating elements for light responsiveness, anaerobic induction, MYB and gibberellin-responsiveness cis-regulating elements in the 2100 bp region upstream of the *AHLs* promoters (Fig. 7). Approximately 43.5% of the selected genes contained a MYB binding sites, and previous studies have shown that the *MYB* gene family can regulate anther development and function formation [35, 36]. In addition, more than 198 and 183 MYB members directly or indirectly involved in responses to drought stress were described in *Arabidopsis* and rice, respectively [37], including a *AHL* gene in rice [22]. However, there are few studies on plant stress and hormone effects of the *AHL* gene family. Therefore, it is possible that the *AHL* gene family can also mediate responses to drought stress in soybean. All selected *AHL* promoters contain the light responsiveness element, suggesting that the *AHL* genes participated in plant light morphogenesis in soybean. Approximately 91.3% of the selected *AHLs* had the anaerobic induction element. Under anaerobic conditions, plant disease resistance is reduced, root morphological formation is imperfect, and root tip epidermal cells are damaged or died, leading to pathogen invasion [38]. Hemoglobin is an intracellular signal of hypoxia in plants, and the amount of symbiotic hemoglobin in legumes is relatively high [39]. Higher plants perceive O_2_ molecules through hemoglobin under anaerobic conditions, and the changes in hemoglobin concentration are regulated by partial pressure of O_2_ pressure [39]. Our results predict that *AHLs* play significant roles in soybean anaerobic induction. Gibberellin plays an important role in the growth cycle of plants, promoting cell division and elongation [40], controlling seed germination and enabling roots formation [41, 42]. 17.4% of the selected *AHLs* include the gibberellin-responsiveness element, whereby *AHLs* may participate in the regulation of growth and development in soybean, confirming the variety of functions played by *AHLs* in soybean growth. Similarly, in the study of grape *AHL* genes, it was found that all grape *AHL* genes contain *cis*-elements related to light response, stress response and hormone response, indicating that not only in soybean, but in other species, *AHL* genes may affect plants growth and development [43].

### Co-expression network analysis of the *AT-hook motif* gene family in soybean

**Fig. 8:**
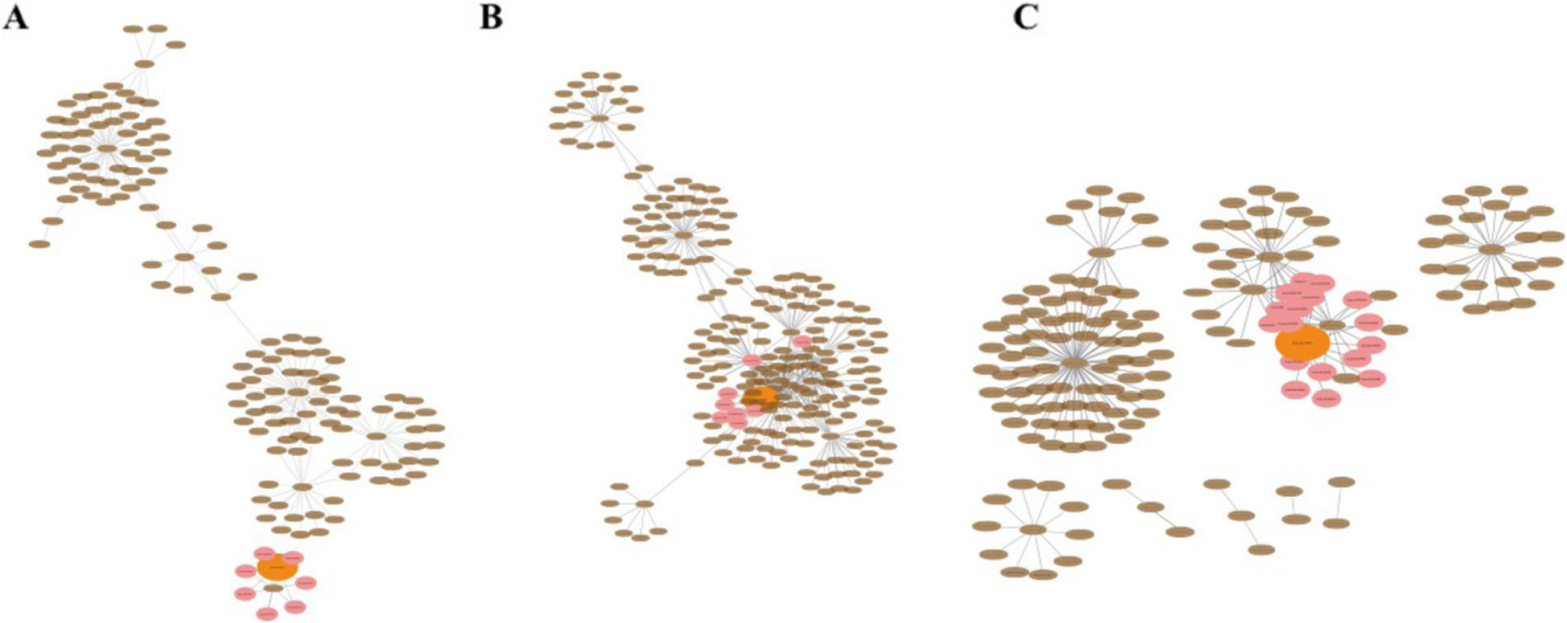
Co-expression network involving in soybean. The whole network for Type-I (**a**), Type-II (**b**) and Type-III (**c**) were drawn with brown ellipses. The genes interacting with AHLs are shown as pink circles, and the selected *AHL* genes correspond to the orange circles

A co-expression network was used to represent the upstream and downstream genes that interact with *AHLs* in the three different Types (Fig. 8). We picked out the representative genes from the co-expression network and the annotated genes functions are available in the supplementary material Table 6. Our study demonstrates that some *AHLs* are associated with genes related to energy binding, such as *Glyma.11G179200 Glyma.09G196600*, that might be involved in soybean energy transduction. The co-expression network indicates that in addition to interacting with other genes, *AT-hook motif* genes also interacted to some extent with each other. For example, Type II *Glyma.20G212200* interacted with four *AT-hook motif* genes to jointly regulate the expression of other genes. We also found that *AT-hook motif* genes are involved in biological processes histone binding and ATP binding in soybean and that the same gene is involved in histone modification in *Arabidopsis thaliana* [17]. In our speculations, part of *AHL* genes is related to nucleation signals and mainly distributed in Type-II, whereby, *AHL* genes regulates the nucleation process of other proteins in soybean. The reported *DELLA (LeGAI)* gene is expressed in both nutritional and reproductive tissues in tomato and this gene family is also involved in GA signal transduction [44]. In our research, that the *AHL* gene of *Glyma.20G212200* was co-expressed with two *Glyma.05G140400* and *Glyma.08 g095800 DELLA* genes. Similarly, *Glyma.16G204400* and *Glyma.08 g095800 Glyma.05G140400 DELLA* genes interact to regulate the gibberellin transduction pathway in soybean. Therefore, we consider that the *AT-hook motif* gene family is involved in gibberellin signal transduction pathway in soybean. Together, our results show that the *AHL* gene family is involved in regulating biological processes such as energy transduction, the gibberellin pathway and the nuclear entry signal pathway in soybean.

**Table 6:** Annotation of genes present in co-expression network

Class	Gene ID	Gene describition	Biology Process
Type-I	**Glyma.14G066800**	AT-hook motif nuclear-localized protein 15	DNA-binding transcription factor activity
	Glyma.09G199800	AP2-like ethylene-responsive transcription factor AIL6	DNA binding
	Glyma.20G095500	DUF724 domain-containing protein 3	histone binding
	**Glyma.17G136600**	AT-hook motif nuclear-localized protein 24	DNA-binding transcription factor activity
	Glyma.01G165000	alpha-mannosidase	alpha-mannosidase activity
	Glyma.09G163500	NA	NA
	Glyma.02G281500	alpha-amylase inhibitor/lipid transfer/seed storage family protein	NA
	Glyma.13G260800	NA	NA
	Glyma.10G128200	HVA22-like protein e	6-phosphofructokinase activity
	Glyma.05G054200	AT-hook motif nuclear-localized protein 24	DNA-binding transcription factor activity
	Glyma.19G118400	WUSCHEL-related homeobox 11	DNA binding
	Glyma.18G063900	NA	NA
	Glyma.09G090600	uncharacterized LOC100790863	NA
	Glyma.14G096300	pyrophosphate--fructose 6-phosphate 1-phosphotransferase subunit beta	6-phosphofructokinase activity
	Glyma.08G080200	berberine bridge enzyme-like 8	FAD binding
	Glyma.03G192700	NA	ATP binding
	Glyma.03G088300	NA	NA
	Glyma.07G170100	NA	NA
	Glyma.17G166500	UDP-glycosyltransferase 84B2	quercetin 3-O-glucosyltransferase activity
	**Glyma.14G028600**	AT-hook motif nuclear-localized protein 16	DNA-binding transcription factor activity
	Glyma.04G005900	5′-methylthioadenosine/S-adenosylhomocysteine nucleosidase 2	catalytic activity
	Glyma.20G212200	AT-hook motif nuclear-localized protein 6	DNA binding
	Glyma.06G005700	5′-methylthioadenosine/S-adenosylhomocysteine nucleosidase 1	catalytic activity
	Glyma.06G005600	5′-methylthioadenosine/S-adenosylhomocysteine nucleosidase 1-like	catalytic activity
	Glyma.10G178000	AT-hook motif nuclear-localized protein 6	DNA binding
	Glyma.09G153600	AT-hook motif nuclear-localized protein 6	DNA binding
	Glyma.16G204400	AT-hook motif nuclear-localized protein 7	DNA binding
	**Glyma.20G039300**	AT-hook motif nuclear-localized protein 28	DNA-binding transcription factor activity
	Glyma.06G130400	NA	NA
	Glyma.08G357100	NA	cell fate determination
	Glyma.09G055200	NA	NA
	Glyma.08G358000	NA	NA
	Glyma.14G182200	NA	NA
	Glyma.03G075700	GDP-mannose transporter GONST3	antiporter activity
	Glyma.09G058200	defensin-like protein 183	Fungicide
	Glyma.20G067800	NA	hydrolase activity
	Glyma.08G350700	putative E3 ubiquitin-protein ligase RING1b	Metal-binding
	Glyma.05G071500	uncharacterized LOC106798883	NA
	Glyma.13G262900	F-box/FBD/LRR-repeat protein At3g14710	F-box domain-containing protein
	Glyma.02G104800	scopoletin glucosyltransferase	UDP-glycosyltransferase activity
	Glyma.19G053600	uncharacterized LOC106797433	mitochondrial cytochrome c oxidase assembly
	Glyma.19G092600	pectinesterase inhibitor-like	pectinesterase inhibitor activity
	Glyma.20G063400	NA	NA
	Glyma.10G125600	NA	NA
	Glyma.10G294000	high mobility group B protein 15	DNA binding
	Glyma.02G113200	NA	transferase activity
	Glyma.08G350700	putative E3 ubiquitin-protein ligase RING1b	Metal-binding
	Glyma.17G188200	beta-glucosidase BoGH3B	beta-glucosidase activity
	Glyma.08G235200	LBD domain-containing transcription factor	LOB domain-containing protein
Type-II	**Glyma.20G212200**	AT-hook motif nuclear-localized protein 6	DNA binding
	Glyma.05G140400	DELLA protein GAI 1	gibberellic acid mediated signaling pathway
	Glyma.02G285500	AT-hook motif nuclear-localized protein 16	DNA-binding transcription factor activity
	Glyma.08G095800	DELLA protein GAI1	gibberellic acid mediated signaling pathway
	Glyma.06G150000	carbamoyl-phosphate synthase small chain, chloroplastic	carbamoyl-phosphate synthase (glutamine-hydrolyzing) activity
	Glyma.14G028600	AT-hook motif nuclear-localized protein 16	DNA-binding transcription factor activity
	Glyma.05G207300	AT-hook motif nuclear-localized protein 5-like	DNA binding
	Glyma.03G011200	AT-hook motif nuclear-localized protein 9	DNA binding
	Glyma.08G014000	AT-hook motif nuclear-localized protein 5	DNA binding
	**Glyma.17G155200**	NA	DNA binding
	Glyma.10G148800	importin subunit alpha-2	NLS-bearing protein import into nucleus
	Glyma.01G219600	AT-hook motif nuclear-localized protein 5	DNA binding
	Glyma.15G144800	importin subunit alpha-2	NLS-bearing protein import into nucleus
	Glyma.05G053800	AT-hook motif nuclear-localized protein 1	DNA binding
	Glyma.09G153600	AT-hook motif nuclear-localized protein 6	DNA binding
	Glyma.09G105600	carbon catabolite repressor protein 4 homolog 1	poly(A)-specific ribonuclease activity
	Glyma.03G207300	carbon catabolite repressor protein 4 homolog 1	poly(A)-specific ribonuclease activity
	Glyma.02G169700	NA	poly(A)-specific ribonuclease activity
	Glyma.05G111800	AT-hook motif nuclear-localized protein 13	DNA binding
	Glyma.19G204800	carbon catabolite repressor protein 4 homolog 1	poly(A)-specific ribonuclease activity
	Glyma.07G072300	AT-hook motif nuclear-localized protein 9	DNA binding
	Glyma.11G042900	AT-hook motif nuclear-localized protein 8	DNA binding
	Glyma.01G198900	AT-hook motif nuclear-localized protein 8	DNA binding
	Glyma.17G136200	AT-hook motif nuclear-localized protein 1	DNA binding
	Glyma.09G260600	uncharacterized LOC100814615	DNA binding
	Glyma.18G231300	AT-hook motif nuclear-localized protein 9	DNA binding
	Glyma.16G204400	AT-hook motif nuclear-localized protein 7	DNA binding
	Glyma.07G153700	BAG family molecular chaperone regulator 4	adenyl-nucleotide exchange factor activity
	Glyma.01G123300	BAG and ubiquitin domain-containing protein	adenyl-nucleotide exchange factor activity
	Glyma.09G039600	mportin subunit alpha-2	NLS-bearing protein import into nucleus
	Glyma.17G031000	importin subunit alpha-2	NLS-bearing protein import into nucleus
	Glyma.03G051600	BAG family molecular chaperone regulator 4	adenyl-nucleotide exchange factor activity
	Glyma.03G208600	uncharacterized LOC102667761	BRCT domain-containing protein
	Glyma.20G239200	importin subunit alpha-2	NLS-bearing protein import into nucleus
	Glyma.06G075100	glucan endo-1,3-beta-glucosidase 5	glucan endo-1,3-beta-D-glucosidase activity
	Glyma.07G132100	kinesin-like protein KIN-10B	ATP binding
	Glyma.11G023900	AT-hook motif nuclear-localized protein 5	DNA binding
Type-III	**Glyma.17G136200**	AT-hook motif nuclear-localized protein 1	DNA binding
	Glyma.03G179700	zinc finger protein JACKDAW	DNA-binding transcription factor activity
	Glyma.13G139000	zinc finger protein JACKDAW	DNA-binding transcription factor activity
	Glyma.12G055600	DNA damage-repair/toleration protein DRT100	NA
	Glyma.10G051500	zinc finger protein JACKDAW	DNA-binding transcription factor activity
	Glyma.19G180400	zinc finger protein JACKDAW	DNA-binding transcription factor activity
	Glyma.17G257500	HVA22-like protein i	NA
	Glyma.18G279800	putative GDSL/SGNH-like acyl-esterase family protein	O-acetyltransferase activity
	Glyma.11G179200	receptor protein kinase TMK1	ATP binding
	Glyma.06G122200	sugar efflux transporter SWEET13	sugar transmembrane transporter activity
	Glyma.09G260600	uncharacterized LOC100814615	DNA binding
	Glyma.11G042900	AT-hook motif nuclear-localized protein 8	DNA binding
	Glyma.05G111800	AT-hook motif nuclear-localized protein 13	DNA binding
	Glyma.07G072300	AT-hook motif nuclear-localized protein 9	DNA binding
	Glyma.17G155200	NA	DNA binding
	**Glyma.16G204400**	AT-hook motif nuclear-localized protein 7	DNA binding
	Glyma.01G198900	AT-hook motif nuclear-localized protein 8	DNA binding
	Glyma.03G011200	AT-hook motif nuclear-localized protein 9	DNA binding
	Glyma.18G231300	AT-hook motif nuclear-localized protein 9	DNA binding
	Glyma.03G258300	auxin response factor 18	auxin-activated signaling pathway
	Glyma.09G243200	uncharacterized LOC100807657	mRNA binding
	Glyma.02G285500	AT-hook motif nuclear-localized protein 16	DNA-binding transcription factor activity
	Glyma.08G095800	DELLA protein GAI1	DNA-binding transcription factor activity
	Glyma.06G164800	mediator of RNA polymerase II transcription subunit 36a	histone-glutamine methyltransferase activity
	Glyma.05G140400	DELLA protein GAI 1	gibberellic acid mediated signaling pathway
	Glyma.14G028600	AT-hook motif nuclear-localized protein 16	DNA-binding transcription factor activity
	Glyma.09G196600	uncharacterized LOC100813911	GTPase activity
	Glyma.04G084200	probable transcriptional regulatory protein At2g25830	NA
	**Glyma.19G249200**	AT-hook motif nuclear-localized protein 14	DNA binding
	Glyma.02G272200	abscisic-aldehyde oxidase	Metal binding
	**Glyma.18G220900**	AT-hook motif nuclear-localized protein 1	DNA binding
	Glyma.09G248700	UPF0510 protein INM02-like	NA
	Glyma.11G225500	UDP-glycosyltransferase 76B1	quercetin 3-O-glucosyltransferase activity
	**Glyma.13G150600**	AT-hook motif nuclear-localized protein 1	DNA binding
	Glyma.13G237200	glyoxysomal processing protease, glyoxysomal	serine-type endopeptidase activity
	Glyma.18G010900	E3 ubiquitin-protein ligase AIRP2	ubiquitin protein ligase activity
	Glyma.06G142100	WRKY transcription factor 55	DNA-binding transcription factor activity
	Glyma.07G201900	FHA domain-containing protein At4g14490	mRNA binding
	Glyma.03G139900	NA	NA
	Glyma.11G059900	cell division cycle-associated 7-like protein	regulation of transcription
	Glyma.17G112700	ABC transporter F family member 4	ATP binding
	Glyma.05G151900	protein RALF-like 24	calcium-mediated signaling
	Glyma.04G139900	U3 small nucleolar ribonucleoprotein protein IMP4-like	snoRNA binding
	Glyma.03G257500	cytochrome b561 and DOMON domain-containing protein At3g61750	NA

### Expression profiles of the *AT-hook motif* gene family in soybean

**Fig. 9:**
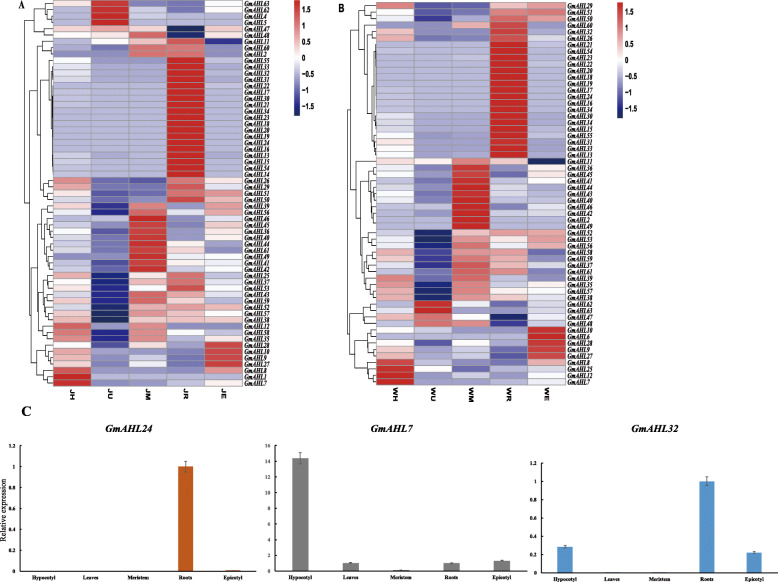
The expression levels of *AT-hook motif* genes in Jack (**a**) and Williams82 (**b**). The colors going from blue to red indicate an increasing level of expression. The cluster tree on the left was classified based on expression levels. The horizontal axis represents the expression level of the same gene in different tissue. The ordinate represents the level of expression of different genes in the same tissue. Tissue specific expression of the *AT-hook motif* genes and expression patterns of three genes in Williams82 (**c**). Expression of *Glyma.05G111500*, *Glyma.20G087200* and *Glyma.06G093400* and in leaves, meristem, roots, epicotyl and hypocotyl at the VC stage. M: Meristem; U: Unifoliate leaves; R: Roots; E: Epicotyl; H: Hypocotyl

To address the expression patterns of the *AT-hook motif* gene family, we selected the representative soybean cultivars, Jack and Williams82 at different tissues and during the VC stage. The transcription data is available from NCBI (accession number: SRP285849) [45]. W82 and Jack were used to investigate whether there were differences in the expression profiles of the *AT-hook motif* gene family between different soybean varieties (Fig. 9a and b). The expression results showed that *AHLs* were mostly expressed in roots and meristems, and that these patterns were similar in W82 and Jack. There are 35 and 31 genes with high expression levels in Jack and W82 roots, respectively. Of the 35 highly expressed genes in Jack’s roots, 22 expressed the same as W82. Of the remaining 13 genes with inconsistent expression, 9 genes had high expression in Jack. In meristem, 26 and 24 genes are highly expressed in Jack and 21 in W82, respectively. The results of the study find that the expression of the same gene differs between different varieties. For example, the expression level of *Glyma.09G260600* is higher in Jack and lower in W82. The expression levels in the leaves of both Jack and W82 are very low, with the exception of 5 genes in Jack and 4 genes in W82. This corroborates previous results in maize [19]. In the Jack’ epicotyl, we find 5 highly expressed genes, similar to W82. In the hypocotyl, *Glyma.04G091600* and *Glyma.06G093400* are both highly expressed, and the expression is consistent. But the expression level of *Glyma.18G036200* of the hypocotyl in W82 is higher than that of Jack. Interestingly, the genes showing high levels of expression in meristematic tissues are mainly distributed in Type-II, while those highly expressed in the roots mainly belong to Type-I. These results indicate that although the *AHL* genes in Jack and W82 had similar expression patterns in different tissues, different genes were expressed differently between the two varieties. Hence, different *AHL* genes may have different functions in the two varieties, and may play important roles in plant development. At the same time, for verification the data of RNA-seq, 3 genes for RT-qPCR were performed to evaluate the expression pattern of three genes in the roots, leaves, meristem, epicotyl and hypocotyl of W82 (Fig. 9c). The results show that it is consistent with the transcriptome.

### The expression of the *AT-hook motif* gene family under drought and submergence

**Fig. 10:**
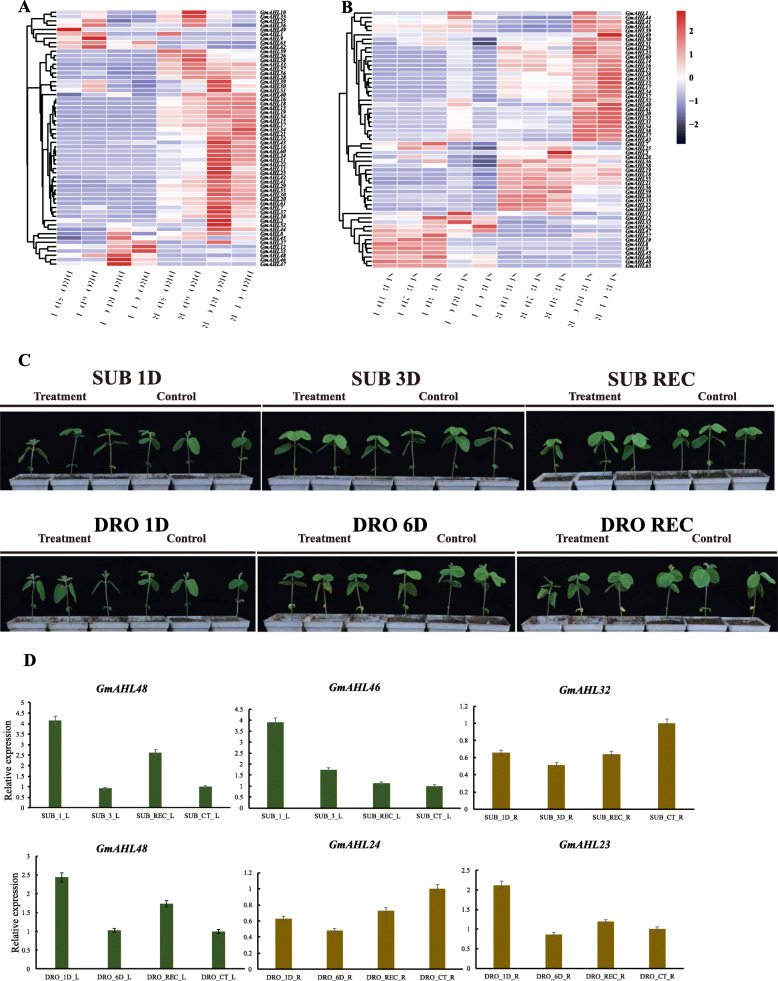
Expression patterns of the *AHL* genes under **a** drought and **b** submergence conditions in Williams82. DRO and SUB represent drought and submergence, respectively. D represents day. CT represents control treatment. L and R are the leaves and roots, respectively. DRO_REC_L/R means 1 day recovery following 6 days of drought in leaves/roots. SUB_REC_L/R means 1 day recovery following 3 days of submergence in leaves/roots. The growth of soybeans under submergence and drought stresses **c**, the left is the treatment group, the right is the control group. Expression of *Glyma.18G231300*, *Glyma.07G072300* and *Glyma.20G087200, Glyma.05G111500* and *Glyma.17G155400* in leaves and roots l at the V1 stage (**d**)

Both drought and submergence have adverse effects on plant growth and a previous study has shown that *AHLs* mediate plant response to drought stress [22]. And in the study of grape *AHLs*, after PEG treatment, the *AHL* genes has different degrees of response to the stress [43]. so we hypothesis that *AHLs* in soybean may also impact in drought stress responses in in soybean. Hence, we tested the expression of genes in the leaves and roots of W82 under submergence and drought conditions (PRJNA574626) at the V1 stage (Fig. 10a and b). The RNA transcription data is from NCBI. Both in the control and treatment showed that a higher number of *AHLs* were expressed in roots compared to the leaves, which is consistent with the results in Fig. 9a and b. After 5–6 days of drought treatment, the expression of highly expressed genes, such as *Glyma.02G285500*, considerably reduced. However, the expression of *Glyma.14G181200* increased, especially after 6 days of drought treatment in leaves. In the roots, drought treatment led a significant reduction of expression genes compared to the control group. Similar patterns were observed under submergence treatment, where some genes, such as *Glyma.14G066800*, showed significantly higher expression in leaves than controls. Overall, the levels of expression of most genes were decreased after submergence in roots.

We used roots and leaves at V1 stage of W82 to verify the expression of *AHL* genes under drought and submergence stresses (Fig. 10d). Our study found that after 1 day of submergence stress, the expression level of *AHL* genes in leaves increased significantly, and the expression decreased significantly after 3 days of submergence. When the treatment was restored for 1 day, the expression level of *AHL* genes were same as that of the control. The expression level in roots decreased after submergence stress. The expression of *AHL* genes increased significantly after 1 day of drought stress, and decreased after 6 days of drought in the leaves. As the stress time increased, the expression level decreased compared with the control in the roots after drought stress. At the same time, we recorded the phenotype of soybean under submergence and drought stress (Fig. 10c). After mannitol stress treatment, the expression of *OsAHL1* was increased at the beginning, and as time increased, the expression of *OsAHL1* began to decrease [22]. As the stress time increases, the soybean plant under stress is shorter and more wilting than the control, but the phenotypic difference is not particularly obvious.

These results suggest that during stress condition, gene expression overall increases in the leaves and decreases in the roots. Furthermore, we also found that after 1 day of recovery, the levels of gene expression were restored, and were sometimes even higher than those of the control group. The different expression patterns indicate that *AHLs* are more expressed in the roots, and are involved in responses to drought and submergence stress.

## Discussion

### Identification of the *AT-hook motif* gene family in soybean

It’s well documented that soybean is the staple crop in world, and provides a great source of proteins for human populations. Previous studies in *Arabidopsis thaliana*, maize and cotton have provided comprehensive information and the basis for our research on soybean, revealing the multiple functions associated with of *AHLs*, particularly involved in regulating plant growth and stress responses [19, 20, 25]. We decided to further study the *AHL* gene family in soybean as this may provide the molecular basis for high-stress tolerance in plants and shed light on the improvement of environmental adaptation.

We identified *AHL* soybean genes from the JGI Phytozome website [46]. These genes were predicted based on the presence of a PPC domain and the AT-hook motif, and were included in the Pfam website [47]. In this study, 63 *AT-hook motif* genes were identified in soybean and generated a phylogenetic tree using the MEGA7 software [48]. According to the phylogenetic tree, the *AT-hook motif* gene family is divided into two Clades on the basis of PPC domain, Clade-A and Clade-B, respectively. Among them, Clade-B is further classified into two Types on the basis of the AT-hook motif, Type-II and Type-III. Clade-A is also referred to as Type-I. That the PPC domain of Clade-A has more changes, which is consistent with the results in maize [19]. Our results indicates that more changes in the PPC domain lead adaptation in plants. The flanking sequences of the AT-hook motif in soybean are similar to other land plants [1], and most AHL genes belonged to Clade-A, whereby this clade seemingly contains richer and more conserved functions that are essential for plant survival. In our paper, the *AHL* gene family was distributed on 18 chromosomes, independently of chromosome size and location. We also found that segmental duplication events are the main form of duplication in the *AHL* gene family in soybean, which contrasts to observations in maize showing dispersive duplication is more common [19]. This illustrates that the *AHL* gene family expanded in different ways in different species.

### Conversation of the *AT-hook motif* gene family in soybean

The *AHL* gene family is conserved across land plants, and all AHL genes share a PPC/DUF domain. In Clade-A, this PPC/DUF domain contains the conserved L-R-S-H motif, while Clade-B displays F-T-P-H. We were also able to observe that the diversity of the *AHL* gene family in soybean extends beyond the amino acid sequences of the PPC/DUF domain and is also present in the AT-hook motif sequences, which have an R-G-R core. However, while the sequence of this core in Clade-A is R-G-R-P in Clade-B it is R-G-R-P-R-K-Y. It has been previously suggested that Clade-B evolved from Clade-A [1]. The gene structures of the *AT-hook motif* gene family with UTR-less and multiple-CDS. Twelve genes in Clade-A show UTR-less. And in Type-II and Type-III, the number of intron is increased. So we speculate that the increase of introns leads to the diversity of protein structures.

The collinearity analysis showed that soybean AHLs have high degrees of homology with other species, as shown by comparisons in four different plant species: *Oryza sativa*, *Zea mays*, *Populus trichocarpa*, *Medicago sativa*.

### Expression patterns in soybean

The expression patterns based on cis-elements found in the promoter regions show that *AHL* genes may participate in plant light morphology, growth and development, and also stress response. Co-expression analysis indicates that AHL proteins may be involved in the gibberellin pathway, which is involved in plant responses to drought and excess water. Previous study has shown that gibberellin can be involved in plant drought and water flooding stress [49]. Overexpression of *CBF/DREB2* in *Arabidopsis thaliana* can reduce the content of active GAs and improve drought tolerance [50], and the CYP96B4/ SD37 in the amycin synthesis pathway is related to the drought tolerance in rice [49]. The drought tolerance of the *dss1* mutant is significantly higher than that of the wild type, which is due to the decrease of GA_1_ [51].

The stress caused by long-term water-flooding in rice inhibits the levels of ethylene, reduces the amount of active GAs, and thus inhibits the elongation of the internodes [52, 53]. It is found that the *AHL* genes may be involved in the gibberellin pathway, and the *AHL* gene family may also regulate the gene expression in response to drought and flood stress in soybean. Therefore, the *AHLs* expression of W82 under drought and flood conditions was analyzed. Our results indicated that, under these stress conditions, the expression of *AHL* genes decreased in the roots. At the same time, the expression of *AHLs* in different tissues from distinct soybean varieties indicated that the expression of *AHLs* was higher in the roots. We also used the W82 leaves and roots of the V1 stage to verify. It is interesting to find that the gene expression levels in the leaves on the first day of stress treatment increased significantly, and then decreased. Regarding the mechanism of this phenomenon, it is also needs further study. In order to further explore the *AHL* gene family, we did a correlation analysis between the number of introns and gene expression level in W82 (Table 7). The analysis showed that in different tissues, except for the roots, the *p* values of other tissues are all less than 0.05 and are positively correlated. Under stress conditions, similarly, the p value of leaves is less than 0.05 and is positively correlated, while roots are not correlated. The specific mechanism has not yet been resolved. In future research, we will further study the molecular mechanism, but it is certain that the number of introns in soybeans does affect the expression of *AHL* genes to a certain extent. Accordingly, the *AHL* gene family plays an important role in soybean resilience, providing a theoretical basis for future breeding of this important crop.

**Table 7:** Correlation between the number of introns in the *AHL* genes and gene expression level

Tissue	*p*-value	Correlation coefficient
Hypocotyl	0.005682	0.3557964
Unifoliate leaves	0.0002118	0.4643242
Meristem	3.68E-11	0.7341764
Epicotyl	0.001313	0.4086413
Roots	0.8362	−0.0274978

## Conclusion

We characterized 63 *AHL* genes in soybean and analyzed their respective motif composition. The phylogenetic tree divided these genes into two clades based on the PPC domain. We also investigated the cis-acting elements of the promoter regions of *AHL* genes and their co-expression network, and systematically studied the *AHLs* expression profiles in different tissues and varieties, as well as the response to stress conditions. The systematic exploration of *AHL* genes in soybean lays the foundation for future work in soybean breeding.

## Methods

### Identification of the *AT-hook motif* gene family

The *AT-hook motif* gene family of *Arabidopsis thaliana* was obtained from the TAIR database (https://www.arabidopsis.org/) [54]. The amino acid sequences of the *AT-hook motif* genes of soybean and sorghum were from JGI Phytozome website (https://phytozome.jgi.doe.gov/pz/portal.html) and Ensemble Plants (https://plants.ensembl.org/index.html) [46, 55]. We used Pfam (https://pfam.xfam.org) to predict the genes containing the PPC domain, and then filtered out the genes containing both the PPC domain and AT-hook motif [47]. The homology comparison of amino acid sequences of *Arabidopsis thaliana*, soybean and sorghum was performed. We used online ExPASy program (http://www.expasy.org/tools/) to determine the biochemistry of each AHL protein, including the number of amino acids, the molecular weight (MW) and predict the isoelectric point (pI) parameters [56].

### Phylogenetic analysis

We used a Neighbor-Joining tree to represent the phylogenetic relationship between the *AHL* genes [57]. The amino acid sequences of *Arabidopsis thaliana*, *Glycine max* and sorghum were selected to construct the phylogenetic tree by using the MEGA7 software [48]. We implemented a total of 1000 bootstraps to present the evolutionary history [58].

### Gene structure analysis

We used MEME (http://meme-suite.org/) to predict the conserved motif of AT-hook motif in the *AHL* gene family with an e-value of 10^− 5^ in soybean [59], and obtained a total of 10 conserved motifs. The final file was generated by TBtools [60]. The gene structure of the *AT-hook motif* genes was analyzed using the TBtools software [60]. The structures of the genes were mapped through CDS and genome sequencing. We used the SMART website (http://smart.embl-heidelberg.de/) to evaluate the accuracy of the selected proteins [61].

### Chromosome location analysis, collinearity analysis and GO annotation analysis

Chromosome mapping information for the *AT-hook motif* genes was obtained from JGI Phytozome Ensemble Plants. The map of chromosome locations was drawn using the TBtools software [60]. We selected full-length amino acids sequences for four species to perform collinearity analysis with soybean. The collinear relationship was estimated using the MCScanx and TBtools software [60, 62]. We used the Soy Base (https://www.soybase.org) website to conduct GO analysis on 63 *AT-hook motif* genes.

### Cis-acting elements analysis and co-expression network

We obtained 2100 bp genome sequences spanning the promoter regions of the *AT-hook motif* gene family of *Glycine max* from NCBI. The cis-acting elements were analyzed using TB tools [60]. Co-expression analysis of the *AT-hook motif* gene family was derived from find new members of a pathway in SoyNet (www.inetbio.org/soynet) [63]. The resulting sif files were downloaded and visualized with Cytoscape to construct the co-expression network [64].

### Expression pattern analysis

The transcription data was obtained from the NCBI database (https://www.ncbi.nlm.nih.gov). We processed the transcriptome data and constructed the heat map in R. The fragments-per-kilobase-per-million (FPKM) value was used to quantify gene expression. The heatmap map was built according to the observed expression levels.

### Quantitative RT-PCR (qRT-PCR) for *AHL* genes

Williams82 was used plant material and grown in a greenhouse 26 °C and 14 h/ 10 h light/dark conditions. The meristem, leaves, epicotyl, hypocotyl and roots were collected separately in the VC stage, with three independent replicates per sample. We did three levels of treatment during the V1stage, control, submergence treatment and drought treatment. Drought treatment for 6 days and rehydrated for 1 day, and the leaves and roots were taken for RNA extraction on the first day, the sixth day, and 1 day after the rehydration. Submergence treatment for 3 days and 1 day for recovery, the leaves and roots were taken for RNA extraction on the first day, the third day and the recovery day. Fresh plant materials were immediately frozen in liquid nitrogen for RNA extraction. We used the SYBR Green I Master mixture (Roche, Basel, Switzerland) as qRT-PCR reagent. The designed qRT-PCR primers are shown in Table 8. The 2^−ΔΔCT^ method was used to calculated the relative gene expression levels [65].

**Table 8:** The primers of qRT-PCR

*GmAHL24*	F	ACCAACGTGGCTTACGAGAG	R	AGAAGGGTCAGGGAAAGGGT
*GmAHL7*	F	TGCTGCTGCAAGGGTTATGC	R	CTCTAACCAACCAATCCCCACA
*GmTUB*	F	TCTTGGACAACGAAGCCATCT	R	TGGTGAGGGACGAAATGATCT
*GmAHL32*	F	TGGGTAACAGTGGTGGTAATG	R	GTGGCCTCCATTAGGGATAAG
*GmAHL48*	F	AGGCAATGACAAGGGGAACAT	R	TGCATGAGTGCATAGCAGGG
*GmAHL46*	F	GTTGTGGTTTAGGGGGCACA	R	ACACCCACAATTCTCAGACACA
*GmAHL23*	F	CAACGTGGCTTACGAGAGGT	R	CGTTCGTTCCAGTGGCTGAA

## Data Availability

The data of sequenced mRNA are available in the National Center of Biotechnology Information (NCBI) under the accession number SRP285849 (https://www.ncbi.nlm.nih.gov/sra/SRP285849) and PRJNA574626 (https://www.ncbi.nlm.nih.gov/bioproject/PRJNA574626/). Seed for *Glycine max* cultivar Williams 82 was obtained from the laboratory at the Northeast Forestry University.
